# CTLA4-Ig interacts with cultured synovial macrophages from rheumatoid arthritis patients and downregulates cytokine production

**DOI:** 10.1186/ar2865

**Published:** 2009-11-23

**Authors:** Maurizio Cutolo, Stefano Soldano, Paola Montagna, Alberto Sulli, Bruno Seriolo, Barbara Villaggio, Pierfranco Triolo, Paolo Clerico, Lamberto Felli, Renata Brizzolara

**Affiliations:** 1 Research Laboratories and Academic Unit of Clinical Rheumatology, Department of Internal Medicine, University of Genova, Viale Benedetto XV, 16132 Genova, Italy; 2 Clinical Academic Unit of Nephrology, Department of Internal Medicine, University of Genova, Viale Benedetto XV, 16132 Genova, Italy; 3 Rheumatoid Arthritis Unit - Orthopedic Surgery Department, CTO Hospital, Via Zuretti 10126 Turin, Italy; 4 Orthopedic Department, Largo Rosanna Benzi, University of Genova, 16132 Genova, Italy

## Abstract

**Introduction:**

Co-stimulatory signal B7(CD80/CD86):CD28 is needed in order to activate T cells in immune response. Cytotoxic T lymphocyte-associated antigen-4-immunoglobulin (CTLA4-Ig) binding to the B7 molecules on antigen-presenting cells downregulates this activation and represents a recent biological treatment in rheumatoid arthritis (RA). Objectives of the study were to investigate the presence of the B7.2 (CD86) molecule and its masking by CTLA4-Ig on cultures of both RA synovial macrophages (RA SM), and of macrophages differentiated from THP-1 cells (M). In addition, the anti-inflammatory effects of CTLA4-Ig on co-cultures of RA SM and M with activated T cells were tested.

**Methods:**

All macrophages were co-cultured for 24 hours with activated T cells, without or with CTLA4-Ig (10, 100, 500 μg/ml for 1 hour, 3 hours and overnight, respectively). Immunofluorescence (IF) staining for B7.2, and an analysis of inflammatory cytokine expression (interleukin (IL) -6, tumor necrosis factor (TNF) α, IL-1β, transforming growth factor (TGF) β) by immunocytochemistry (ICC), western blot (WB) and reverse transcriptase-polymerase chain reaction (RT-PCR) were performed.

**Results:**

Macrophages showed intense B7.2 expression. CTLA4-Ig/B7.2 masking was evident for all macrophages, even after only 1 hour of cell culture (range from 10 to 100 μg/ml). ICC of co-cultures showed a dose-dependent decrease in inflammatory cytokines (*P* < 0.001 for IL-6, TNFα, IL-1β and TGFβ). Data were confirmed by WB and RT-PCR analysis.

**Conclusions:**

Optimal concentrations of CTLA4-Ig for the CTLA4-Ig/B7.2 masking on activated macrophages were identified and were found to induce significant downregulation in the cell production of IL-6, TNFα, IL1-β and TGFβ. In conclusion, macrophages would appear to be a sensitive target for CTLA4-Ig treatment in RA.

## Introduction

Rheumatoid arthritis (RA) is a prototype of an immune-mediated chronic inflammatory disease and is considered a model for studying and validating new targeted biological therapies.

Migration of activated lymphocytes and monocytes into the synovial tissue in RA is one of the first steps in synovial inflammation, followed by subsequent damage of other joint components [1-3]. In recent years, the role of T cells has regained some importance in the immunopathology of RA, thus providing a rationale for the specific targeting of T cells with biologic treatments [4-8].

T cell response is triggered by an initial signal delivered through the T cell receptor (TCR), and it recognizes the antigenic peptide within the context of the major histocompatibility complex (MHC) molecule on the antigen-presenting cells (APC). In order to be fully activated, it needs to be followed by another signal, which is provided by the signals of the co-stimulatory molecules that are expressed on APC (such as dendritic cells, B-lymphocytes and macrophages) [9].

Among the known multiple co-stimulatory signals, one of the best described is the CD80/CD86:CD28 pathway [10,11]. CD80 (B7.1) and CD86 (B7.2), which are expressed on APC, bind the CD28 molecule on the T cells, thereby transducing the co-stimulatory signal in the early phase of the immune response. However, the activated T cells then express the cytotoxic T lymphocyte-associated antigen-4 (CTLA-4) molecule, which binds the B7 molecules on APC with a 10- to 20-fold greater affinity compared with CD28, and downregulates the T cell activation [12-14].

CTLA-4-Ig, a biological agent, is constructed by genetically fusing the external domain of human CTLA-4 and a fragment of the Fc domain of human immunoglobulin G1 (IgG1), which has been modified to be non-complement fixing. Like the native CTLA-4, the fusion protein (CTLA-4-Ig) binds more avidly to CD80/CD86 (APC) than to CD28 (T cells), thus interfering with CD28/B7 interaction [15,16].

Therefore, taking these mechanisms into consideration, several randomized, double-blind, placebo-controlled clinical trials have demonstrated that CTLA-4-Ig improves the signs and symptoms of RA in patients with inadequate response to methotrexate or/and anti-TNF agents [17-20].

However, because macrophages play a crucial role in various steps of the synovial RA pathophysiology, the aim of this *in vitro *study was firstly, to search for the presence of the B7.2 molecule on the surface of cultured synovial macrophages (SM) obtained from active RA patients, and then to investigate the modulatory effects of CTLA-4-Ig in a co-culture of RA SM or macrophages together with an activated T cell line [21]. In particular, the investigation focused on the effects of CTLA-4-Ig on the production of peculiar mediators of inflammation produced by macrophages, such as cytokines (IL-6, TNFα, IL-1β) and transforming growth factor beta (TGFβ).

## Materials and methods

### Rheumatoid arthritis synovial macrophages (RA SM) cultures

RA SM were obtained from seven patients (five females and two males, mean age: 47 ± 12 years, disease duration 4 ± 6 years, Disease Activity Score using 28 joint counts (DAS28) >5.2) who fulfilled the 1987 revised criteria of the American College of Rheumatology for adult RA and who underwent therapeutic arthroscopic synoviectomy or knee replacement surgery.

At the time of surgery, all patients had been taking non-steroidal anti-inflammatory drugs for 20 days and low-dose glucocorticoids (prednisone range 5 to 7.5 mg/day) for three or four months. No intra-articular treatments, systemic biological drugs, antiproliferative drugs or other disease modifying anti-rheumatic drugs (DMARDs) were being administered at the time of knee surgery, nor had they been for at least four months prior to it. The Ethics Committee of the University of Genova approved the study and informed consent was obtained from all patients.

After surgery, the synovial RA tissue samples were carefully cut into small pieces (2 to 5 mm), washed in Dulbecco's phosphate buffered saline (DPBS; Sigma-Aldrich, Sigma Chemical Division, Milan, Italy) and incubated in collagenase (0.75 mg/ml; type IV from *Clostridium histolyticum*; Sigma-Aldrich, Sigma Chemical Division, Milan, Italy) for one hour at 37°C. The digest was passed through a pore size 58 Å ~150 mesh wire to separate the synovial cells from the debris tissue. The cells were then washed three times with DPBS, resuspended in RPMI-1640 medium (Sigma-Aldrich, Sigma Chemical Division, Milan, Italy) that had been supplemented with 10% fetal bovine serum (containing < 0.5 EU/ml endotoxin), 2 mmol/l L-glutamine, 100 μg/ml streptomycin, and 100 U/ml penicillin (Sigma-Aldrich, Sigma Chemical Division, Milan, Italy). Viability of the cells (90 to 95%) was tested by trypan blue exclusion.

The synovial cells were seeded into flexiperm chamber slides (International, PBI S.p.a. Milan, Italy; 105 cells/well) and cultured in 5% CO_2_ air humidified atmosphere at 37°C. After one hour, non-adherent cells were washed out, while adherent cells (RA SM) were incubated in culture medium in the presence or in the absence of lipopolysaccharide (LPS) stimulus (10 μg/ml) for one hour to investigate B7.2 expression [22].

B7.1 expression was not tested because it is expressed in lower amounts on resting cells and is only upregulated with prolonged T cell stimulation, while B7.2 is constitutively expressed and rapidly upregulated on APC with an antigen-specific signal, thus indicating that B7.2 is chiefly involved in mediating initial T cell activation [10,23].

EBV+ transfected human B-lymphocytes were also incubated in flexiperm chamber slides as a positive cell line control for B7.2 expression.

### THP-1 cell-line cultures

THP-1 (cell bank. Interlab Cell Line Collection. Clinical Pathology Dept, IST, Genova, Italia) human monocytes were incubated with phorbol myristate acetate (PMA; 0.5 μg/ml) for two hours to differentiate into macrophages, then cells were seeded into flexiperm chamber slides (International, PBI S.p.a. Milan, Italy; 10^5^ cells/well) and cultured in 5% CO_2_ air humidified atmosphere at 37°C.

### Macrophage and T cell co-cultures

#### Macrophage/Jurkat

The macrophages that were obtained from THP-1 as previously described, were co-cultured with human T cells for 24 hours (Jurkat T cells previously activated with concanavalin-A (5 μg/ml) for 20 hours). They were then seeded into multiwell flat bottom plates both in the absence and the presence of various concentrations of CTLA-4-Ig (10, 100, 500 μg/ml). The macrophage/T cell ratio was 1:1.

At the end of the co-culture incubation period, the T cells were removed with several washes in PBS. Macrophages were harvested, washed in PBS, and used for the various assays. Preliminary immunocytostaining using a specific monoclonal antibody to identify the macrophages (anti-human HAM56, Dako, Carpinteria, CA, USA) was performed. All experiments were performed in triplicate.

#### RA SM/Jurkat

RA SM that had been isolated from synovial tissue as previously described, were co-cultured into multiwell flat bottom plates with T cells (Jurkat T cells previously activated with concanavalin-A (5 μg/ml) for 20 hours) for 24 hours, both in the absence and presence of CTLA-4-Ig at various concentrations (100, 500 μg/ml). The *in vitro *CTLA-4-Ig concentrations to be tested were chosen on the basis of the available literature, suggesting values that are very close to the therapeutic doses used to treat RA [22,24,25]. The macrophage/T cell ratio was 1:1.

At the end of the co-culture incubation period, the T cells were removed with several washes in PBS. The RA SM were harvested, washed in PBS, and used for the various test assays. In order to identify macrophages, we performed preliminary immunocytostaining with a specific monoclonal antibody (anti-human HAM56, Dako, Carpinteria, CA, USA). All experiments were performed in triplicate.

### Immunofluorescence assay

Slides with cellular spots obtained from cultures of macrophages were seeded into flexiperm chamber slides, then fixed in acetone for 30 seconds, air dried, and rehydratated in PBS. The spots were then incubated with an fluorescein isothiocyanate anti-human CD86 (B7.2) mouse antibody (BD, Biosciences, New York, NY, USA) at room temperature for 45 minutes, washed in PBS, and cover slipped with glycerol medium. Evaluation of the fluorescence expression for CD86 (B7.2) was performed by fluorescence microscopy (Leica, Cambridge, UK).

### Immunocytochemistry assay

Macrophages from co-cultures that had been seeded into multiwell flat bottom plates were harvested mechanically and incubated on glass slides for 45 minutes at 4°C, then the cellular spots were fixed in acetone for 30 seconds, air dried, and stored at -20°C until use.

After rehydration in PBS, the slides were incubated in a 3% H_2_O_2 _solution for 15 minutes at room temperature to prevent non-specific reaction due to endogenous peroxidases.

The spots were then incubated with anti-cytokine (anti-IL6, anti-TNFα, anti-IL-1β) and anti-TGFβ monoclonal antibodies (all diluted 1:100 at room temperature for 45 minutes. Santa Cruz Biotechnology, Santa Cruz, CA, USA).

Linked antibodies were detected by a biotinylated universal (pan-specific) antibody and subsequently by horseradish peroxidase streptavidin (Vector Laboratories, Burlingame, CA, USA). Each step was followed by two washes in PBS. The staining reaction was developed by the diaminobenzidine system (DakoCytomation, Dako North America, Inc., Carpinteria, CA, USA). Finally, slides were counter-stained with hematoxylin, fixed with ethanol, and cover-slipped with Eukitt mounting medium for microscope preparations (O. Kindler GmbH, Friburg, Germany). Negative controls were treated the same way, except that the primary antibodies were omitted.

Image analysis of immunocytochemistry slides was performed using the Leica Q-Win image analysis system (Leica, Cambridge, UK). Approximately 100 cells were analysed for each sample, and pixels/mm^2 ^(positive area) were quantified by the Leica Q-Win software (Leica, Cambridge, UK). The individual cells were randomly selected by the operators using the cursor and were then automatically measured as positive areas.

### Immune enzymatic assay (ELISA)

After 24 hours of treatment, the culture medium was harvested and stored at -20°C until analysis. The enzymatic immunoassay for quantitative determination of IL-6, TNFα and IL-1β was carried out with a microplate kit system (Diaclone, Besançon, France). The results were obtained with a multiwell plate automatic processor (Techno Genetics, Milan, Italy).

### Western blot analysis

After the various treatments, macrophage pellets were lysed in a buffer containing 20 mmol/l Tris-HCl pH 8, 150 mmol/l NaCl, 1 mmol/l phenylmethylsulphonyl fluoride, 5 mg/ml aprotinin, and 0.5% Nonidet P-40 (Promega, Milan, Italy) for one hour at 4°C. The lysates were then centrifuged for 10 minutes at 13,000 rpm. Protein samples (20 mg) were diluted with sample buffer and separated by 10% SDS-PAGE. The proteins were transferred to a Hibond-C nitrocellulose membrane (GeHealthCare Europe, Friburg, Germany), after which the membrane was blocked for one hour at room temperature in PBS containing 5% non-fat powdered milk.

To carry out immunoblot analysis, the membrane was incubated with rabbit anti-IL-6 (diluted 1:500), goat anti-TNFα (1:200) and goat anti-TGFβ (1:200) antibodies (Santa Cruz Biotechnology, Santa Cruz, CA, USA) overnight at 4°C. Then membranes were washed in 1% PBS + 0.05% Tween 20, pH 7.4 and incubated for one hour at room temperature with a secondary anti-rabbit antibody for IL-6 (1:5,000; Santa Cruz Biotechnology, Santa Cruz, CA, USA) and a secondary anti-goat antibody for TNFα and TGFβ (1:65,000; Sigma, Milan, Italy). After three further washes with PBS/Tween, a bound secondary antibody was detected through emitting chemiluminescent reaction (Immobilon, Millipore, CA, USA).

Western blot analysis was not performed on RA SM because it would have been difficult to obtain enough cells from the primary culture to allow full analysis of the cytokines being investigated.

Densitometry analysis of the western blot bands were performed by Delta Sistemi Analysis Software (version 3.0.02, Latina, Italy).

### Reverse transcriptase-polymerase chain reaction

mRNA extraction from the various experimental conditions of macrophage/Jurkat co-cultures was performed with the Rneasy Mini Kit (Qiagen, Valencia, CA, USA).

The samples were analysed by RT-PCR, using both IL-6-, TNFα-, TGFβ-specific primers (Invitrogen S.R.L., Milan, Italy) and beta-actin-specific primers (Promega, Milan, Italy) as the internal positive control. Statistical analysis was carried out by the non-parametric Wilcoxon T test.

Statistical analysis was performed to compare the paired treatments. *P *< 0.05 was considered statistically significant.

## Results

### B7.2 (CD86) positivity on cultured macrophages

**Figure 1:**
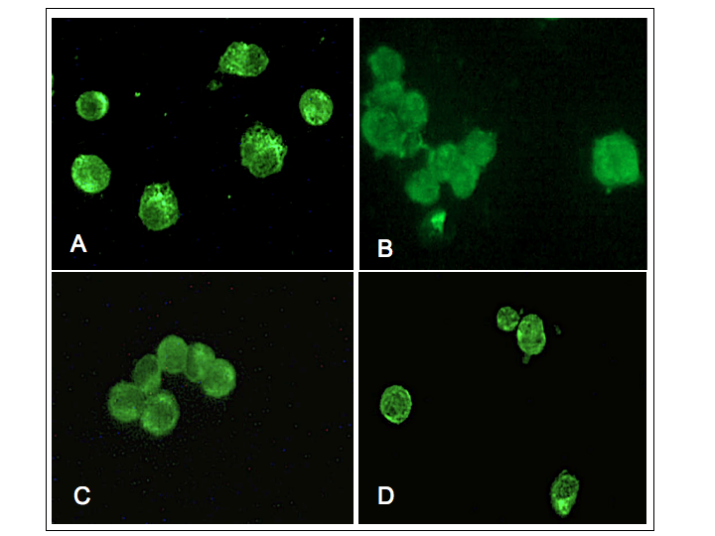
B7.2 expression on macrophages by immunofluorescence analysis. B7.2 expression by immunofluorescence analysis **(a) **on primary cultures of rheumatoid arthritis synovial macrophages (RA SM) untreated, **(b) **on macrophages differentiated from THP-1 untreated, **(c) **on SM pre-treated with lipopolysaccharide and **(d) **on EBV+ B lymphocytes (positive control cell line).

Macrophages showed intense positivity for B7.2 and a diffuse distribution pattern on the cell surface both in RA SM cultures and in macrophage cultures. No differences were found between LPS-treated or untreated cells, and no differences were found between cultured macrophages and the positive control cell line either (EBV+ transfected human B-lymphocytes; Figure 1).

### *In vitro *CTLA-4-Ig/B7.2 masking on macrophages

Analysis by both fluorescence and optic microscopy in light field showed a reduction of B7.2 positivity on macrophages treated with CTLA-4-Ig, certainly due to CTLA4-Ig binding to B7.2 and subsequent B7.2 expression masking.

**Figure 2:**
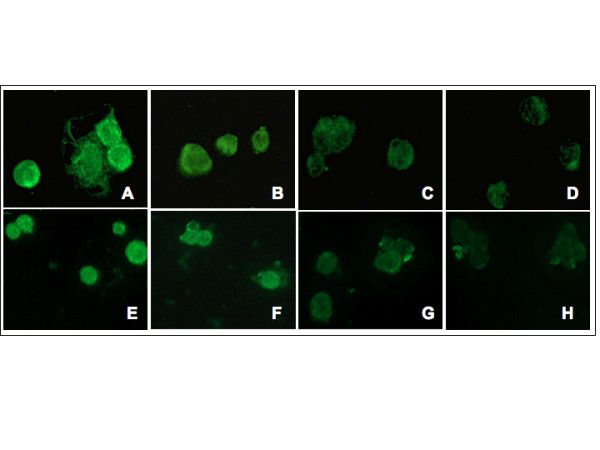
B7.2 expression in macrophages after CTLA4-IG treatment by immunofluorescence analysis. B7.2 expression by immunofluorescence analysis on primary cultures of rheumatoid arthritis synovial macrophages (RA SM) **(a) **untreated, **(b) **treated with CTLA4-Ig 10 μg/ml, **(c) **treated with CTLA4-Ig 100 μg/ml and **(d) **treated with CTLA4-Ig 500 μg/ml; **(e) **on macrophages differentiated from THP-1 untreated, **(f) **treated with CTLA4-Ig 10 μg/ml, **(g) **CTLA4-Ig 100 μg/ml and **(h) **CTLA4-Ig 500 μg/ml.

The positive staining of B7.2 showed a gradual decrease from CTLA-4-Ig-untreated macrophages (controls) to CTLA-4-Ig-treated macrophages (from 10 to 500 μg/ml; Figure 2). B7.2 masking on macrophages was still evident within the CTLA-4-Ig range from 10 to 100 μg/ml after one hour.

### Effects of CTLA-4-Ig on cytokine expression in macrophage and T cell co-cultures

#### Macrophage/Jurkat

**Figure 3:**
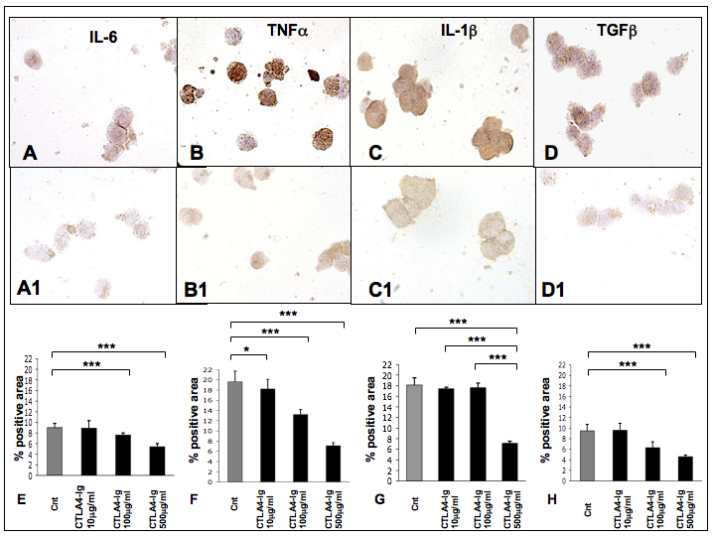
Immunocytochemistry for IL-6, TNFα, IL-1β and TGFβ expression in co-cultures of macrophage/Jurkat. Immunocytochemistry in co-cultures of macrophage/Jurkat, **(a) **untreated (controls) and **(a1) **treated with CTLA4-Ig 500 μg/ml for IL-6, **(b) **untreated (controls) and **(b1) **treated with TNFalpha, **(c) **untreated (controls) and **(c1) **treated with IL-1β and **(d) **untreated (controls) and **(d1) **treated with TGFβ. Quantification (mean value ± standard deviation) of immunocytochemistry (ICC-QWin) for **(e) **IL-6, **(f) **TNFα, **(g) **IL-1β and **(h) **TGFβ expression in co-cultures of macrophage/Jurkat, untreated (controls) and treated with CTLA4-Ig (10, 100, 500 μg/ml). * *P *< 0.05; ** *P *< 0.01; *** *P *< 0.001.

Macrophages that had been co-cultured with T cells following the addition of CTLA-4-Ig (range from 100 to 500 μg/ml), showed a decrease in pro-inflammatory cytokine content, as evaluated by immunocytochemistry (Figures 3a to 3d). The cytokines we evaluated were IL-6, TNFα, IL-1β and TGFβ, as growth factor.

The changes that were observed in IL-6, TNFα and TGFβ expression included a significant, dose-dependent decrease (*P *< 0.001), following treatment with CTLA-4-Ig at both 100 and 500 μg/ml. IL-1beta expression only showed a significant decrease for CTLA-4-Ig (500 μg/ml) treatment (*P *< 0.001). Interestingly, as compared with untreated macrophages (controls), we found a significant decrease (*P *< 0.05) which was limited to TNFα expression, even after administering only 10 μg/ml of CTLA-4-Ig (Figures 3e to 3h).

**Figure 4:**
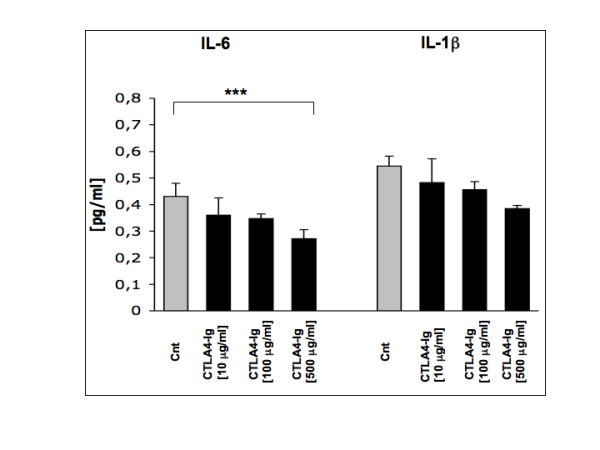
Evaluation of cytokine production by ELISA assay. Evaluation by ELISA assay of IL-6 and IL-1β production in supernatants of co-cultured macrophage/Jurkat untreated (controls (cnt)) and treated with CTLA4-Ig (10, 100, 500 μg/ml). Results are expressed as mean value ± standard deviation from three experiments. * *P *< 0.05; ** *P *< 0.01; *** *P *< 0.001.

ELISA for IL-6 and IL-1β confirmed a slight, dose-dependent reduction in cytokine production in the media of co-cultures after treatment with CTLA-4-Ig as compared with untreated cells (Figure 4). Interestingly, we were unable to dose TNFα concentrations in culture medium because they were beyond the standard curve, even following a 1:10 diluition of the culture medium.

**Figure 5:**
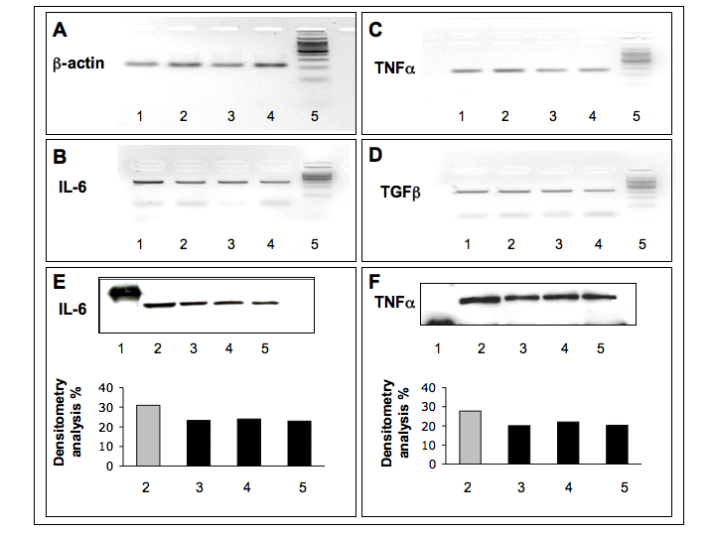
Reverse transcriptase-polymerase chain reaction and western blot analysis for IL-6, TNFα and TGFβ expression in co-cultures of macrophage/Jurkat. Reverse transcriptase-polymerase chain reaction assay in co-cultures of macrophage/Jurkat, untreated (line 1: control) and treated with CTLA4-Ig 10 μg/ml (line 2), CTLA4-Ig 100 μg/ml (line 3), CTLA4-Ig 500 μg/ml (line 4), for **(a) **β-actin (internal positive control), **(b) **IL-6, **(c) **TNFα and **(d) **TGFβ. Line 5: molecular weight. Densitometry analysis of western blot in co-cultures of macrophage/Jurkat, untreated (line 2: control) and treated with CTLA4-Ig 10 μg/ml (line 3), CTLA4-Ig 100 μg/ml (line 4), CTLA4-Ig 500 μg/ml (line 5) for **(e) **IL-6 and **(f) **TNFα protein expression in co-cultures of macrophage/Jurkat. Line 1: molecular weight.

Analysis of mRNA expression in macrophages treated with CTLA-4-Ig versus untreated macrophages (controls) showed a downregulation of cytokine production for IL-6, TNFα and TGFβ (Figures 5a to 5d). Western blot results by densitometry analysis showed a slight, overall decrease in IL-6 and TNFα protein expression (Figures 5e and 5f) in CTLA-4-Ig-treated cells versus untreated cells, while TGFβ levels were not detectable.

#### RA SM/Jurkat

**Figure 6:**
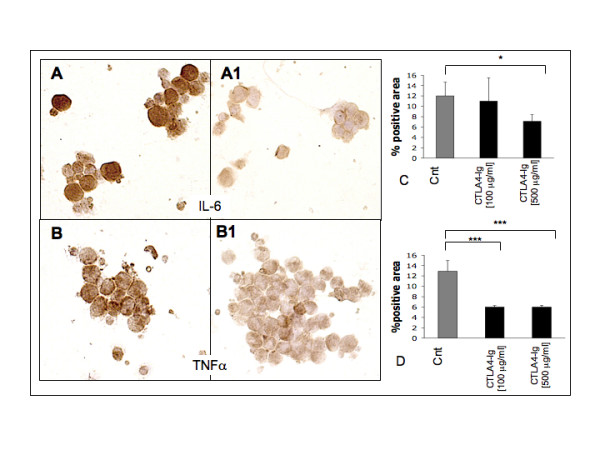
Immunocytochemistry for IL-6 and TNFα expression in co-cultures of RA SM/Jurkat. Immunocytochemistry in co-cultures of rheumatoid arthritis synovial macrophages (RA SM)/Jurkat, **(a) **untreated (controls) and **(a1) **treated with CTLA4-Ig 500 μg/ml for IL-6 and **(b) **untreated (controls) and **(b1) **treated with CTLA4-Ig 500 μg/ml for TNFα. Quantification (mean value ± standard deviation) of immunocytochemistry (ICC-QWin) for **(c) **IL-6 and **(d) **TNFα. * *P *< 0.05; ** *P *< 0.01; *** *P *< 0.001.

RA SM that were co-cultured with T cells following the addition of CTLA-4-Ig (range from 100 to 500 μg/ml) induced a decrease in the pro-inflammatory cytokine expression (Figures 6a and 6b). In particular, IL-6 and TNFα showed a significant downregulation following the addition of CTLA-4-Ig at 500 μg/ml (*P *< 0.05 and *P *< 0.001, respectively). Of note, in RA SM and T cell co-cultures, adding CTLA-4-Ig at a lower concentration (100 μg/ml) induced a significant modulation in TNFα expression alone (*P *< 0.001; Figures 6c and 6d).

TGFβ did not show any change in expression following the addition of CTLA-4-Ig (range from 100 to 500 μg/ml) compared with untreated RA SM (controls; data not shown).

## Discussion

The current report is the first to describe the positivity for the B7.2 co-stimulatory molecule in RA SM and its interaction with the CTLA-4 competitor (CTLA-4-Ig).

Earlier studies had already detected the B7.2 molecule within the RA synovial tissue (while B7.1 was found to be almost altogether absent), and particularly so in areas enriched with CD68-positive macrophages, such as the lining layer and close to CD3-positive T cells [23]. However, the real implications of this observation were not determined at that time. Researchers merely believed that it suggested the presence of important therapeutic targets, such as co-stimulatory molecules (B7 complex), in the synovial tissue, at least as far as the early intervention on the immune response in RA was concerned.

The present study shows that the CTLA-4-Ig fusion protein is able to downregulate SM activation (co-cultured with T cells) by interacting with the B7.2 molecule expressed on their surface and by decreasing their production of inflammatory RA cytokines (IL-6, TNFα, IL-1β).

Furthermore, for the first time, the present study shows that CTLA4-Ig treatment reduces the expression of TGFbeta in human macrophages.

Recent reports suggest that TGFβ-induced effects have an autocrine pathogenetic importance in RA; for example, in inducing matrix metalloproteinase-mediated matrix degradation/remodeling. It has also been suggested that in the context of an inflammatory cytokine milieu, TGFβ supports *de novo *differentiation of IL-17-producing T cells. Therefore, these observations may be strengthened by the present results [26,27].

Finally, we must now consider SM as possible direct targets of CTLA-4-Ig-mediated downregulation. As a matter of fact, current concepts suggest that inhibiting T cell co-stimulation is the key mode of CTLA-4 action.

However, additional mechanisms of action may be related to the fact that CTLA-4 directly binds to APC (such as macrophages) and might directly modulate their function. Thus, CTLA-4 might not only indirectly affect T cell interaction with APC, but CTLA-4 may also directly affect its primary cellular target, which is the cells of the monocytic lineage (i.e. macrophages) that strongly express the B7-2 molecule (CD86) in RA synovial tissue.

As is well known, macrophages are not only one of the main inflammatory cytokine-producing cells in the RA synovial tissue, but they also differentiate into bone resorbing osteoclasts, which are implicated in RA inflammatory bone erosions and joint damage [28,29]. Recently, it was found that CTLA-4-Ig directly inhibits the Receptor Activator for Nuclear Factor k B Ligand (RANKL) - as well as the TNFα-mediated osteoclastogenesis *in vitro *in a dose-dependent manner (which is clearly evident and significant at 100 μg/ml), without the need for the concomitant presence of T cells [30].

Furthermore, CTLA-4-Ig was effective at inhibiting the TNF-induced osteoclast formation in a non-T cell-dependent TNF-induced model of arthritis, as well as at inhibiting the formation of inflammatory bone erosions *in vivo *[30].

Therefore, even if CTLA-4-Ig typically works by inhibiting T cell co-stimulation and T cell activation, we must now consider that a further primary cellular target of CTLA-4-Ig might also be mononuclear APC, such as SM that express the B.7 complex.

Although we co-cultured macrophages with T cells, the results clearly suggest direct downregulation of the CTLA-4-Ig fusion protein on the RA SM pro-inflammatory function. The next step is to test these effects on SM monocultures.

Interestingly, recent concepts, such as reverse signalling, have defined that the cell-cell interactions can be rather bidirectional, which means that the 'ligand'-expressing cell (i.e. macrophage) undergoes a functional change upon engaging with the receptor [31]. For example, the binding of CTLA-4 to its ligand B7 might lead to a functional change in APC, as already observed [32,33].

A different approach was previously used to study the role of the CTLA-4 molecule in the inflammatory reaction of RA joints. It involved blocking CTLA-4 with an anti-CTLA-4 antibody and then assessing its effects on TNFα and IL-1 production in synovial fluid mononuclear cell cultures [34]. As expected, adding the anti-CTLA-4 antibody enhanced TNFα and IL-1 production in a dose-dependent manner.

## Conclusions

In conclusion, the CTLA-4-Ig interaction we observed with SM and their subsequent downregulation further support the key role they play in various steps of RA, and may explain the beneficial effects of CTLA-4-Ig fusion protein treatment in controlling the signs and symptoms of RA, even in advanced phases of the disease.

## Abbreviations

APC: antigen-presenting cells; CTLA-4: cytotoxic T lymphocyte-associated antigen-4; DMARDs: disease-modifying antirheumatic drugs; DPBS: Dulbecco's phosphate buffered saline; ELISA: enzyme-linked immunosorbent assay; IgG1: immunoglobulin G1; IL: interleukin; LPS: lipopolysaccharide; MHC: major histocompatibility complex; PMA: phorbol myristate acetate; RA: rheumatoid arthritis; RT-PCR: reverse transcriptase-polymerase chain reaction; SM: synovial macrophages; TCR: T cell receptor; TGF: transforming growth factor; TNF: tumor necrosis factor.

## Competing interests

The authors declare that they have no competing interests.

## Authors' contributions

MC defined the design and coordination of the study, participated in interpretation of data, drafted the manuscript and provided general supervision and final approval of the version to be published. SS participated in the design and coordination of the study and carried out western blot analysis. PM participated in the design and coordination of the study, performed the statistical analysis, cultured the cells and carried out RT-PCR analysis. AS participated in patient selection and clinical evaluation of data. BS participated in patient selection and clinical evaluation of data. BV participated in acquisition and data collection. PT provided surgical tissue samples (synovial tissues) in order to culture the RA SM. PC provided surgical tissue samples (synovial tissues) in order to culture the RA SM. LF provided surgical tissue samples (synovial tissues) in order to culture the RA SM. RB participated in the design and coordination of the study, cultured the cells, carried out immunocytochemistry analysis and helped draft the manuscript.
